# Concomitant bilateral elastofibroma in the infrascapular and gluteal regions: a report of a rare case

**DOI:** 10.1186/s12891-020-3037-7

**Published:** 2020-01-08

**Authors:** Omran Al Dandan, Ali Hassan, Mona Al Muhaish, Jumanah AlMatrouk, Haidar Almuhanna, Tarek Hegazi

**Affiliations:** Department of Radiology, King Fahd Hospital of the University, Imam Abdulrahman Bin Faisal University, Al-Khobar, Saudi Arabia

**Keywords:** Bilateral, Elastofibroma, Gluteal, Infrascapular

## Abstract

**Background:**

Elastofibroma is a benign soft tissue tumor characterized by the presence of elastic fibers in a stroma of collagen and mature adipose tissue. It is reported to have a prevalence of 2.73%, as shown by a study through computed tomography (CT) images. However, multiple elastofibromas are uncommon.

**Case presentation:**

We report a case of concomitant bilateral elastofibroma in the infrascapular and gluteal regions. A 63-year-old male patient presented with a 6-month history of gradually increasing painless swellings in the upper back. On physical examination, firm, painless bilateral infrascapular masses were identified; these masses were more noticeable on forward arm flexion. Contrast-enhanced computed tomography showed well-defined bilateral infrascapular masses deep to the serratus anterior muscles as well as poorly defined bilateral gluteal masses with attenuation similar to that of the adjacent skeletal muscle. Magnetic resonance imaging revealed heterogenous masses with internal fatty streaks, consistent with elastofibroma. The histopathological diagnosis of elastofibroma was established based on the results of image-guided core-needle biopsy. The patient underwent surgical excision of both infrascapular elastofibromas with no post-operative complications. As the gluteal masses were incidental, surgical management was not warranted.

**Conclusion:**

The presence of multiple elastofibromas is unusual. This report describes a rare case of multiple elastofibromas and its typical imaging features, and alerts us that elastofibromas are not exclusive to the periscapular region.

## Background

Elastofibroma is a benign soft tissue tumor characterized by the presence of elastic fibers in a stroma of collagen and mature adipose tissue. This tumor was first reported in 1961 in the 12th Histopathology Scandinavian Congress by Järvi and Saxen who briefly described four cases of this tumor and called it elastofibroma [1].

Although previously believed to be rare, elastofibroma has a prevalence of 2.73%, as shown by a large retrospective study through computed tomography (CT) images [2]. Autopsy studies have reported an even higher prevalence of up to 24% in the elderly population [3]. However, multiple elastofibromas are uncommon. Herein, we present the first case of concomitant bilateral elastofibroma in the periscapular and gluteal regions in a 63-year-old male patient.

## Case presentation

We report a case of a 63-year-old man who presented with a 6-month history of a gradually increasing swelling on the right upper back. Although the swelling was painless, the patient experienced some discomfort because of its size. He had also observed a comparatively smaller swelling on the left side of the same region. Furthermore, he recently experienced unintentional weight loss. He reported no history of heavy manual work, trauma, or malignancy during his lifetime, and his past medical history was unremarkable.

Physical examination revealed the presence of a palpable, non-tender, firm mass with limited mobility located beneath the scapular tip of each shoulder. However, it was more prominent on the right side. The masses were more noticeable on forward arm flexion. No palpable lymph nodes were noted, and the back and shoulder exhibited a normal range of motion. Routine laboratory investigations showed no abnormal findings.

**Fig. 1:**
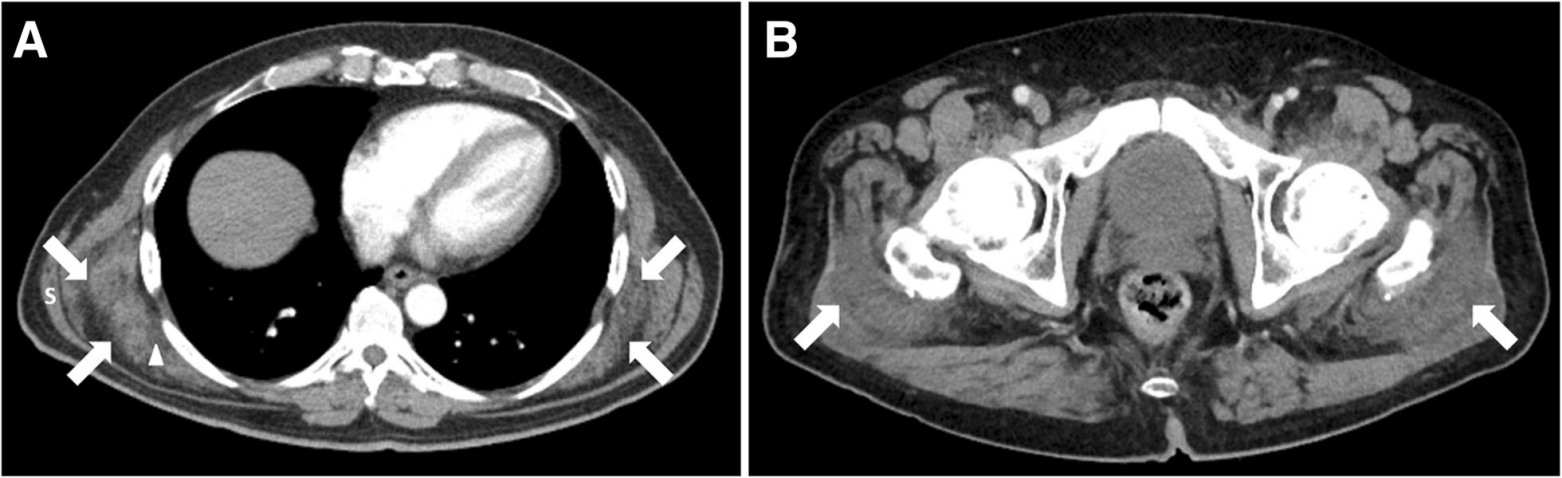
Axial contrast-enhanced CT images showing bilateral well-defined unencapsulated masses within the infrascapular regions (arrows in **a**), deep to the serratus anterior muscles (S) with attenuation similar to that of the adjacent skeletal muscle and internal areas of streaky fat (arrowhead). In addition, there are similar appearing bilateral poorly defined soft tissue masses (arrows in **b**) at the level of the greater trochanters

The patient underwent contrast-enhanced CT, which showed well-defined, bilateral, solid masses located deep to the serratus anterior muscles in the infrascapular region (Fig. 1a). The right and left infrascapular lesions measured 9 × 3 × 6 cm and 7 × 2 × 5 cm, respectively. The masses appeared isodense relative to the surrounding skeletal muscles and exhibited a multilayered appearance with internal fatty streaks. No invasion or associated bony lesions were observed. Moreover, two soft tissue masses were bilaterally present between the gluteus maximus and gluteus minimus muscles (Fig. 1b), measuring 6 × 3 × 5 cm and 7 × 2 × 6 cm on the right and left sides, respectively. They were predominantly located at the level of the greater trochanters. The imaging characteristics were similar to those of the infrascapular lesions.

**Fig. 2:**
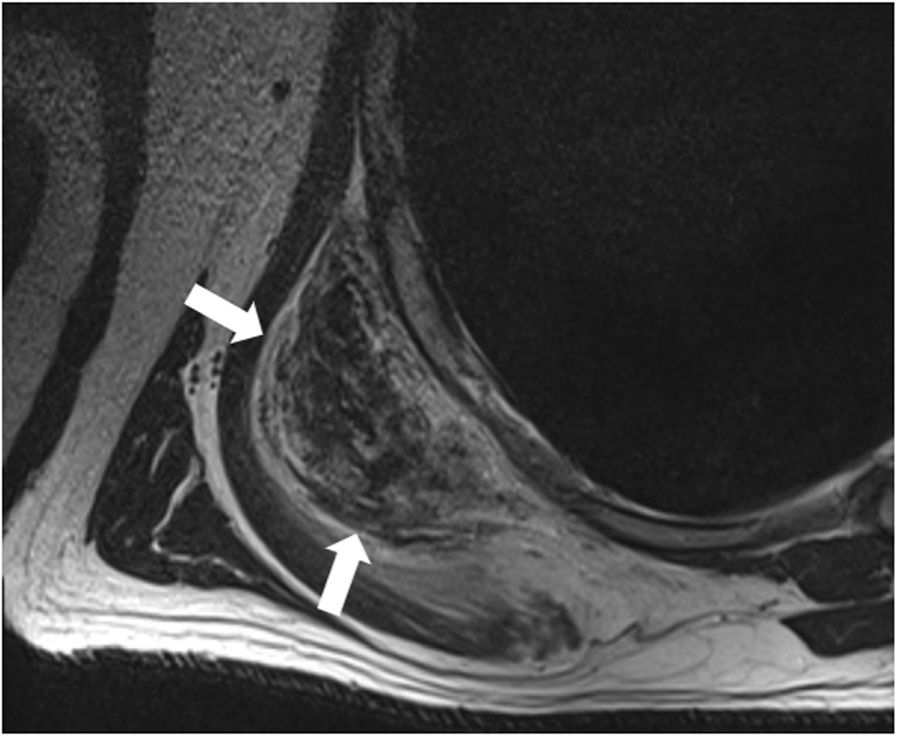
Axial T2-weighted turbo spin-echo (TSE) MR image showing a well-defined mass within the right infrascapular region (arrows) exhibiting multilayered appearance with internal fatty streaks consistent with elastofibroma

Magnetic resonance imaging (MRI) was performed using a 1.5-Tesla scanner, and T1- and T2-weighted axial and coronal scans were acquired without contrast administration. The bilateral infrascapular lesions showed muscle-like signal intensity on both sequences (Fig. 2). Focal linear hyperintensities were observed within the masses following the signal intensity of fat, with no signs of local infiltration.

**Fig. 3:**
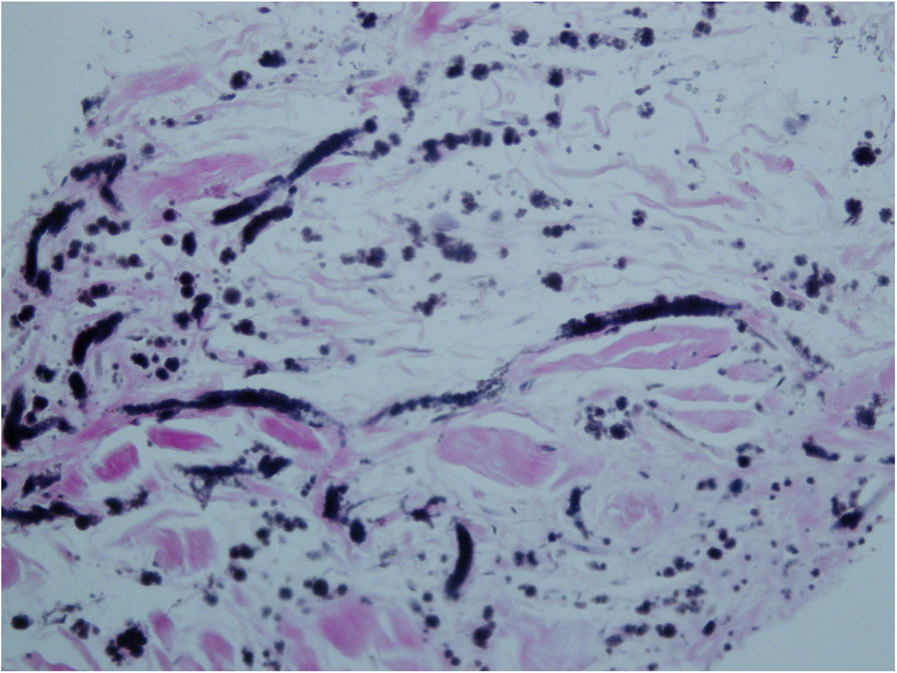
Elastic stain reveals thick elastic cylinders and globules (black) with fragmentation and beading alternated by collagen bundles that is consistent with the diagnosis of elastofibroma

On the basis of the clinical and radiological appearances of the infrascapular and gluteal masses, a presumptive diagnosis of elastofibroma was made. However, considering the patient’s anxiety and history of weight loss, biopsy was recommended during a multidisciplinary oncology meeting. A histopathological diagnosis of elastofibroma was established through core-needle biopsy of the right infrascapular mass (Fig. 3).

The diagnosis was discussed with the patient, and he decided to undergo surgical resection of the infrascapular masses. However, the gluteal masses were asymptomatic and did not warrant surgical management.

Both infrascapular masses were completely excised. The masses were not encapsulated and exhibited a rubbery consistency. Histopathological analysis revealed the presence of fibrous collagenous tissue, occasional fibroblasts, and a large number of eosinophilic fibers intermixed with islets of mature adipose tissues. The resected lesions had clear surgical margins. At the follow-up visit, the patient was asymptomatic and showed no functional limitation in the movement of the shoulder joint.

## Discussion and conclusion

Elastofibroma is often observed beyond the sixth decade of life [4, 5]. Although extremely rare, it has also been reported in children and in infants [6, 7]. This tumor has female predilection. Nagamine et al. studied 170 cases of elastofibroma, of which only 12 cases were in males [5].

The classic site of elastofibroma is the scapular region between the sixth and the eighth ribs and the inferior angle of the scapula, beneath the serratus anterior, latissimus dorsi, and levator scapulae muscles [5]. It is more frequently observed on the right side, but bilateral lesions are common [5, 8]. Rarely, elastofibroma may occur in different anatomic locations causing diagnostic difficulties, including the deltoid muscle, thoracic wall, gluteal and inguinal region, axilla, hand, foot, elbow, orbit and some visceral organs such as stomach, omentum and rectum [9, 10]. The size of elastofibroma varies. In their cohort of 61 cases of elastofibroma, Deveci et al. have reported that the average diameter of the elastofibroma was 8.7 cm [11].

Besides concomitant infrascapular and infra-olecranon elastofibromas, multiple elastofibromas are uncommon with few cases reported in the English medical literature [5]. Nagamine et al. reported a cases of infrascapular elastofibroma with involvement of ischial tuberosity [5]. Hassouna et al. reported a case of triple elastofibromas with one in the usual infrascapular region and the other two in the unusual suprascapular region [12]. Olchowy et al. reported a similar case of triple elastofibromas with MRI documentation [13]. Nishida et al. reported a case of elastofibroma that occurred in both thighs and infrascapular regions [14]. Cevolani et al. reported a case of bilateral elastofibroma in the gluteal region [15]. To our knowledge, concomitant bilateral elastofibroma in the scapular and gluteal regions has not been reported previously.

Different environmental and genetic explanations have been postulated to explain the development of elastofibroma; however, the exact pathogenesis remains unclear. It has been suggested that elastofibroma develops due to repetitive mechanical trauma, which induces reactive hyperproliferation and degeneration of fibroelastic tissue [16]. The advanced age of patients with elastofibroma, the higher frequency among manual workers, and the right-hand side predominance support this theory. However, previous studies have also reported that elastofibromas occur in areas exposed to limited mechanical friction and that they can occur in both the dominant and non-dominant sides. Because of these discrepancies, the mechanical theory is insufficient to explain the development of elastofibroma. Giebel et al. have proposed vascular insufficiency as the cause for the degenerative changes [17]. It has been suggested that elastofibroma originates via a very slow neoplastic process; some studies have shown that a monoclonal proliferation with genomic instability is involved in elastofibroma development [18]. Genetic predisposition is a possible explanation because family history of elastofibroma has been reported in up to one-third of studied cases [5, 19]. Elastofibroma has a multifactorial etiology as no single theory has provided an explanation for all cases.

In most cases, elastofibroma is asymptomatic and is diagnosed incidentally. Few cases may present with palpable masses, discomfort, snapping of the scapula, or functional limitations. Symptoms caused by impingement of the brachial plexus may also be observed [20].

Imaging is essential and may preclude the need for biopsy, particularly if the lesions occur at their typical locations and have the typical imaging features [21]. Recognition of elastofibroma is important as the differential diagnoses include primary or secondary sarcoma, neurofibroma, desmoid tumors, and malignant histiocytofibroma [22].

Plain radiographs are usually normal, but they may show a raised scapula or a soft tissue density. Ultrasound shows alternating patterns of hypo- and hyperechoic lines parallel to the chest wall with no significant vascular flow on Color Doppler [23]. CT scans show a non-encapsulated heterogenous soft tissue mass with a density similar to that of skeletal muscles with possible infiltrating hypodense areas representing fat. MRI demonstrates a lenticular-shaped mass with intermediate signal intensity, similar to that of skeletal muscles, on both T1- and T2-weighted images. Linear strands of high signal intensity are seen reflecting the histological composition of adipose tissues within the fibrous mass that suppress in signal on the fat saturated sequences. Although CT and MRI can readily make the diagnosis, avid contrast enhancement or marked dominance of adipose tissue may result in false diagnosis [24]. Positron emission tomography frequently demonstrates mild to moderate uptake of fluorodeoxyglucose [25]. This uptake is related to high vascularity, fibroelastic proliferation, and inflammatory process within the mass. If imaging appearance was atypical or there is any doubt on the diagnosis, either close follow up imaging or biopsy are reasonable recommendations [24]. Although the imaging findings in the present case were typical for elastofibroma, a biopsy was done considering the patient’s anxiety.

Because elastofibroma is an incidental finding in most cases, surgical treatment is often not required and regular follow-up is not needed as malignant transformation has not been previously reported [4]. Complete surgical excision is advised only for symptomatic cases or for cosmetic concerns [21]. Hematoma formation is a possible post-operative complication because of the high vascularity of the periscapular region [4]. Wide and radical resections should be avoided as marginal resection is sufficient [16]. Surgery is curative and local recurrence is unusual and was attributed due to incomplete excision as the borders of the lesion are often indistinct [26].

This report describes a rare case of multiple elastofibromas and its typical imaging features; our findings demonstrate that elastofibromas are not exclusive to the periscapular region.

## Data Availability

Not applicable.

## Abbreviations

CT: Computed Tomography
MRI: Magnetic Resonance Imaging
